# Who Lives Where and Does It Matter? Changes in the Health Profiles of Older People Living in Long Term Care and the Community over Two Decades in a High Income Country

**DOI:** 10.1371/journal.pone.0161705

**Published:** 2016-09-02

**Authors:** Fiona E. Matthews, Holly Bennett, Raphael Wittenberg, Carol Jagger, Tom Dening, Carol Brayne, Cognitive Function, Ageing Studies (CFAS) collaboration

**Affiliations:** 1 MRC Biostatistics Unit, Cambridge Biomedical Campus, Cambridge Institute of Public Health, Forvie Site, Robinson Way, Cambridge, England; 2 Newcastle University Institute of Health & Society, Newcastle University, Edwardson Building, Campus for Ageing and Vitality, Newcastle upon Tyne, England; 3 Department of Public Health and Primary Care, Cambridge Institute of Public Health, University of Cambridge School of Clinical Medicine, Cambridge, England; 4 Personal Social Services Research Unit, London School of Economics and Political Science, Houghton Street, London, England; 5 Institute of Mental Health, University of Nottingham, Triumph Road, Nottingham, England; Cardiff University, UNITED KINGDOM

## Abstract

**Background:**

There have been fundamental shifts in the attitude towards, access to and nature of long term care in high income countries. The proportion and profile of the older population living in such settings varies according to social, cultural, and economic characteristics as well as governmental policies. Changes in the profiles of people in different settings are important for policy makers and care providers. Although details will differ, how change occurs across time is important to all, including lower and middle income countries developing policies themselves. Here change is examined across two decades in England.

**Methods and Findings:**

Using the two Cognitive Function and Ageing Studies (CFAS I: 77% response, CFAS II: 56% response), two population based studies of older people carried out in the same areas conducted two decades apart, the study diagnosis of dementia using the Automated Geriatric Examination for Computer Assisted Taxonomy, health and wellbeing were examined, focusing on long term care. The proportion of individuals with three or more health conditions increased for everyone living in long term care between CFAS I (47.6%, 95% CI: 42.3–53.1) and CFAS II (62.7%, 95% CI: 54.8–70.0) and was consistently higher in those without dementia compared to those with dementia in both studies. Functional impairment measured by activities of daily living increased in assisted living facilities from 48% (95% CI: 44%-52%) to 67% (95% CI: 62%-71%).

**Conclusions:**

Health profiles of residents in long term care have changed dramatically over time. Dementia prevalence and reporting multiple health conditions have increased. Receiving care in the community puts pressure on unpaid carers and formal services; these results have implications for policies about supporting people at home as well as for service provision within long term care including quality of care, health management, cost, and the development of a skilled, caring, and informed workforce.

## Introduction

Long term care changes as the population of a country ages. As the cost of long term care increases and welfare systems have withdrawn from supporting long term care provision as well as closure of long stay hospitals, admission to long term care becomes more income dependent. This differs between countries. Why individuals move into long term care or other types of accommodation is a major concern as many populations are experiencing rapid ageing in the very old. The changes experienced over time in high income countries may be informative for lower and middle income countries with emerging ageing profiles. In the UK successive governments have promoted care and help in the community with the result that older people now remain in their own homes for longer[1]. Although the UK older population is itself ageing, the proportion of the population living in long term care has, in fact, decreased over the last two decades following an earlier increase [2, 3]. Policies that influence the proportion of people who enter long term care are likely to affect the characteristics of residents in such settings and their needs will also change. Caring for those living in the community can, and often does, place major demands on unpaid carers and costs for local authorities whilst some older people continue to require housing that includes care due to cognitive or functional impairment [4–6]. Older people in the UK live in many types of accommodation ranging from being in their own homes to staffed long term care where they receive 24 hour care. Between these is a category of “assisted living facilities”, housing encompassing warden assisted housing and “granny flats”, the latter being self-contained living areas attached to ordinary homes. In these settings the person is not completely independent but considerably less support is provided in general than in long term care (please see Box 1).

**Box 1: Description of places of residence**

- Long term care: Residential homes, nursing homes and long stay hospitals
- Assisted living facilities: Warden assisted flats and “granny flats”
- Community: Independent house or flat

Changes in the profile of residents in long term care will impact on other types of residence as well. It would also be expected that some people with high levels of need will remain resident in the community or in assisted living facilities if the threshold for admission to long term care is raised and it is harder to receive support for moving to long term care.

Differences in the definition of health and social care make it difficult to compare the investment in care between countries but the use of long term care and assisted living facilities raises a series of policy issues relevant to all [7]. This includes the regulation of the quality of care, financial oversight of the care market, commissioning and negotiating care home fees, workforce retention, primary and community health care for care home residents, eligibility criteria for publicly financed care, financing, and means-testing. Decisions on this range of complex policy issues should be informed by adequate robust data.

There are few reports in the literature that can compare the characteristics of people living in long term care or assisted living facilities with those in the community and those that do focus on physical function and health [8, 9]. Population based studies provide an opportunity to extend such work to provide a balanced description. The Cognitive Function and Ageing Studies (CFAS I and CFAS II) hold methodology steady so that comparisons over two decades can be made. The reduction in the proportion of people living in long term care and the increase in prevalence of dementia in such settings (CFAS I: 56%, CFAS II: 69%) have already been reported [10]. The aim of this paper is to compare the cognitive, functional, health, and social characteristics of older people by place of residence and how these have changed over the last twenty years in a high income country with a changing policy environment.

## Method

CFAS I and II are population based cohort studies randomly sampled from the Family Health Service Authority lists, stratified by age group (65–74 years and 75 years and over). CFAS II was a repeat of CFAS I in three of the original sites—Newcastle, Nottingham, and Cambridgeshire. Ethical approval has been continuous and was obtained for CFAS I from a local research committee for each study centre (Cambridge City Research Ethics Committee, East Cambridge and Fenland LREC, Newcastle and North Tyneside Local Research Ethics committee 1, Nottingham Research Ethics committee 1) and from the NRES Committee East of England—Cambridge South Ref: 05/MRE05/37. For CFAS II ethical approval from local research committees was obtained (NHS North of Tyne, Nottinghamshire County Teaching PCT, Cambridgeshire Community Services NHS Trust). Informed written consent was given by all eligible participant or a proxy where appropriate. Baseline fieldwork was conducted over a three year period from 1991 for CFAS I (77% response rate) [11] and 2008 for CFAS II (56% response rate) [12]. Informed consent was sought from the participant or if they themselves were unable from a consultee who was either a family member, carer or someone nominated by the participant, complying with the mental capacity act regulations.

Participants had face-to-face computerised interviews and interviews with informants were requested for a weighted subsample. Informants were nominated by the individual and included family, friends, and formal carers. Importantly for the potentially frail population information on health and characteristics could be supplemented in the case of item non-response.

Both studies used the Automated Geriatric Examination for Computer Assisted Taxonomy (AGECAT) to provide a study diagnosis of dementia and depression. In CFAS I 19% of the baseline screening sample identified as possibly having dementia were assessed for dementia diagnosis. In CFAS II the screen and assessment were combined so that all individuals received the diagnostic assessment. If the key data for the algorithmic approach were not available a diagnostician reviewed information available from the interview, respondent, informant, and observer to create a DSM III R diagnosis using the same process for CFAS I and II.

The modified Townsend scale was used to determine the participant’s independence in activities of daily living (ADL) and instrumental ADL (IADL). An individual is classed as having severe functional impairment if they need help to either wash all over, make a cooked meal, put on shoes or socks or cannot get around outside of their house, if an individual cannot carry heavy bags or do heavy housework then they are perceived to be mild to moderately impaired. When they were able to perform all these tasks without help they were considered not to have functional impairment [13]. Health conditions other than dementia were self-reported and included angina, peripheral vascular disease, hypertension, hypotension, cancer, diabetes, Parkinson’s disease, stroke, heart attack, fits or epilepsy, breathing difficulties, arthritis, headaches, peptic ulcers, anaemia, transient ischaemic attack, current thyroid problems, hearing difficulties, visual impairment, meningitis, and shingles. The Rose scale was used for peripheral vascular disease in CFAS I. Responses for all health conditions other than dementia were combined to calculate a total and categorised into 0, 1, 2, or ≥3 conditions. Marital status (married, not married), years in education (≤9, 10–11, ≥12), self-reported diagnosis of depression (previously diagnosed with depression, not previously diagnosed with depression), loneliness (feels lonely, does not feel lonely), reported friendships (reports having friends, does not report having friends), frequency of seeing relatives (at least weekly, less than weekly), and amount of exercise (no exercise, any exercise) were also self-reported but exercise information was only available in CFAS II and was only in those not chair or bed bound. Being chair or bed bound was interviewer rated.

Part of the CFAS design was to collect place of residence so there were few missing data, although housing categories differed between CFAS I and II. The use of “granny flats” was rare in the 1980s so was not separated from community living; other accommodation groupings remained the same. Individuals were considered as living in the community if they were living in their own home; assisted living facilities if they were in warden assisted accommodation (CFAS I and CFAS II) or in granny flats (CFAS II); and long term care if they were in residential homes, nursing homes, or long stay hospitals.

Estimates were calculated using inverse probability weights to account for sampling design and non-response. There were two weights used for CFAS I. Screening weights adjusted for the oversampling of those aged 75 years or older, age, gender, and deprivation. Assessment weights in addition adjusted for the two stage design. CFAS II weighting adjusted for the same factors as the CFAS I screening weights but also included long term care attendance. It was possible to adjust for long term care attendance in CFAS II because a complete enumeration of the care homes in each of the centres was identified. Differences between CFAS I and CFAS II were reported on the relative size of the change to the overall percentage. A formal hypothesis test of these changes using a logistic regression was conducted where possible but due to item non-response this was not always possible. In the cases where formal testing could occur, the logistic regression was adjusted for age, gender and cognitive status and exact P-values were given when ≤0.1 and reported as >0.1 if not. Sensitivity analyses were conducted in CFAS II to see if there were any differences in the prevalence estimates when a) long term care was not in the weights, and b) for the different classification of “granny flats” by including “granny flats” in community living in CFAS II rather than assisted living facilities.

## Results

### Long term care

#### Overall

The proportion of people living in long term care dropped from 5% to 3% between studies. Over time the proportion of women in long term care did not change, remaining at three quarters of the long term care population (Table 1). Although approximately 80% of those in long term care were aged ≥80 in both studies, there were more aged ≥90 in CFAS II compared to CFAS I (Table 1). Level of education changed from CFAS I to CFAS II with decreases in less than 9 years of education and increases in higher levels (Table 1). A small proportion of long term care residents were still married and this increased slightly from CFAS I (12%) to CFAS II (18%). As reported previously, the estimated prevalence of dementia in long term care was higher in CFAS II (69%) compared to CFAS I (56%). Many more residents in CFAS II (63%) than CFAS I (48%) had three or more different health conditions. Nearly all residents at both time points had functional impairment (Table 1, p>0.1), and more were chair or bed bound in CFAS II (34%) than CFAS I (22%), though this difference was accounted for after adjustment for cognitive status (p = 0.1). Two thirds of those who were not chair or bed bound reported that they did no exercise in CFAS II. There were small increases in self-reported depression and small decreases in depression diagnosed using the AGECAT algorithm (Table 1). Whilst loneliness remained at the same levels, there were small increases in the report of seeing relatives at least weekly and of friendships between the two studies (Table 1, all p>0.1).

**Table 1:** CFAS I and CFAS II demographic percentages for people living in the community, in assisted living facilities and in long term care weighted (W) for age structure, gender and deprivation with 95% confidence intervals at baseline. Measurements from assessment interview also weighted for two stage design in CFAS I. Education and health conditions supplemented by informant interview in CFAS I and CFAS II, functional impairment, relatives, friendships and self-reported depression supplemented in CFAS II. No exercise only measured in those not chair or bed bound.

		*CFAS I*									*CFAS II*								
		Long term care (n = 346, 5%)			Assisted living facilities (n = 683, 9%)			Community (n = 6599, 86%)			Long term care (n = 197, 3%)			Assisted living facilities (n = 484, 7%)			Community (n = 7115, 90%)		
		n	W %	95% CI	n	W %	95% CI	n	W %	95% CI	n	W %	95% CI	n	W %	95% CI	n	W %	95% CI
Female		264	77.3	72.6–81.3	475	70.3	66.8–73.6	3846	58.9	57.7–60.0	141	75.5	68.9–81.2	304	64.7	60.2–68.8	3801	54.7	53.5–55.9
Age	≤69	13	3.4	2.0–5.7	64	8.9	7.0–11.2	1904	27.9	26.9–29.0	11	6.4	3.2–12.4	36	6.5	4.7–8.9	1892	24.9	23.9–25.9
	70–74	24	6.4	4.3–9.4	123	17.3	14.7–20.3	1628	24.3	23.3–25.4	7	2.9	1.4–6.1	82	15.0	12.2–18.3	1784	23.9	22.9–24.9
	75–79	35	9.5	6.9–13.0	172	24.6	21.5–27.9	1516	23.0	22.0–24.0	27	12.7	8.6–18.4	89	17.2	14.1–20.8	1508	21.1	20.1–22.0
	80–84	100	28.2	23.7–33.2	199	29.5	26.2–33.1	1009	15.8	15.0–16.8	38	16.6	12.0–22.5	117	23.8	20.2–27.8	1135	16.9	16.0–17.8
	85–89	101	29.0	24.5–34.1	94	14.3	11.9–17.2	418	6.8	6.2–7.4	59	27.3	21.4–34.2	109	23.1	19.5–27.2	599	9.2	8.5–9.9
	≥90	73	23.5	19.2–28.5	31	5.4	3.8–7.5	124	2.2	1.8–2.6	55	34.1	27.0–42.0	51	14.4	11.0–18.6	197	4.1	3.5–4.7
Married		29	11.8	8.3–16.5	188	27.2	24.0–30.7	3548	53.4	52.2–54.6	36	17.5	12.5–24.0	81	15.8	12.8–19.3	4291	59.0	57.8–60.2
Years in education	≤9	215	74.2	68.8–79.0	550	81.4	78.3–84.2	4819	73.5	72.4–74.5	88	58.0	49.7–65.9	220	48.2	43.6–52.8	1746	26.4	25.4–27.5
	10–11	44	15.8	11.9–20.6	91	13.4	11.0–16.2	1108	16.9	16.0–17.8	46	25.5	19.1–33.1	190	38.8	34.4–43.3	3689	51.5	50.3–52.7
	≥12	29	10.0	7.0–14.0	35	5.2	3.8–7.2	632	9.7	9.0–10.4	26	16.6	11.3–23.7	63	13.0	10.3–16.4	1578	22.1	21.1–23.1
Functional impairment		290	92.8	89.3–95.2	322	48.2	44.4–51.9	1701	26.6	25.5–27.7	*104*	*94*.*0*	88.3–97.0	296	66.5	62.0–70.7	2100	32.1	31.0–33.2
Chair/bedbound		48	21.9	16.9–28.0	6	0.9	0.4–2.1	45	0.7	0.5–0.9	57	33.5	26.3–41.6	13	2.9	1.7–4.9	63	1.0	0.8–1.3
No Exercise		-	-	-	-	-	-	-	-	-	*39*	*66*.*6*	53.6–77.5	58	13.4	10.5–17.1	301	4.7	4.2–5.3
See relatives at least weekly		145	70.0	63.4–75.9	495	77.7	74.3–80.8	4834	77.9	76.8–78.9	113	73.6	65.6–80.3	330	75.6	71.3–79.4	5142	77.6	76.5–78.5
Reported friendships		144	62.7	56.3–68.8	589	87.0	84.2–89.3	5307	80.9	79.9–81.8	110	68.9	61.2–75.6	418	88.4	85.2–91.0	6013	85.3	84.5–86.2
Number of health conditions	0	63	18.5	14.7–23.0	52	7.5	5.7–9.7	724	10.9	10.2–11.7	11	5.5	2.9–10.1	13	2.9	1.7–5.1	492	6.8	6.3–7.5
	1	53	15.9	12.3–20.2	126	18.5	15.7–21.6	1481	22.4	21.4–23.4	25	13.2	8.7–19.6	50	10.1	7.7–13.1	1107	15.5	14.6–16.3
	2	60	18.1	14.3–22.6	149	22.0	19.0–25.3	1507	22.9	21.9–23.9	33	18.5	12.9–25.9	72	15.1	12.1–18.7	1512	21.3	20.4–22.3
	≥3	158	47.6	42.3–53.1	356	52.1	48.3–55.8	2885	43.9	42.7–45.1	119	62.7	54.8–70.0	335	71.9	67.6–75.8	3904	56.4	55.2–57.5
Self-reported depression		24	10.7	7.2–15.5	86	12.5	10.2–15.2	774	11.7	11.0–12.5	17	13.1	8.0–20.7	50	10.3	7.8–13.4	512	7.4	6.8–8.1
**Measurements from assessment interview**																			
Dementia		128	56.4	45.9–66.3	34	9.7	6.4–14.3	164	5.7	4.4–7.3	137	69.3	61.8–76.0	35	8.5	5.9–11.9	302	5.1	4.5–5.7
Depression		33	17.0	11.7–24.1	14	9.5	4.5–18.8	140	8.5	6.7–10.9	24	13.5	9.1–19.7	56	11.5	8.9–14.6	453	6.4	5.8–7.0
Loneliness		*32*	*32*.*3*	20.0–47.7	38	24.8	15.8–36.7	233	20.3	17.1–24.1	*33*	*35*.*5*	26.1–46.2	141	30.9	26.8–35.4	1074	16.0	15.2–16.9

Italics indicate some non-response—see Table 2

#### Characteristics by dementia status

Table 2 provides the characteristics of people living in long term care by dementia status. The decrease in those with less than 9 years of education was mainly seen in those with dementia, but was also seen to a lesser extent in those without dementia (Table 2). Those with dementia who were functionally impaired were all severely impaired in both studies, though the level of severe functional impairment was still high in those without dementia (CFAS I 83%, CFAS II 80%, p>0.1). A higher proportion of individuals without dementia had three or more health conditions compared to individuals with dementia in CFAS I and CFAS II (Table 2). Prevalence of self-reported depression in those with dementia remained at 11% in both studies but in those without dementia self-reported depression increased from 5% to 16% between the studies. Small increases in the prevalence of loneliness were seen between CFAS I and CFAS II for those with and without dementia (Table 2, both p>0.1). Although in CFAS I slightly less people in long term care reported seeing their relatives weekly than in CFAS II, in both studies there was no difference by dementia status (Table 2). Fewer people with dementia who were able to respond reported having friends compared to people without dementia in both CFAS I (p = 0.04) and CFAS II (p = 0.03).

**Table 2:** Long term care demographic percentages in CFAS I and CFAS II split for dementia and weighted for age structure, gender and deprivation in CFAS I and CFAS II and additionally for the two stage design in CFAS I with 95% confidence intervals at baseline. Education and health conditions supplemented by informant interview in CFAS I and CFAS II, functional impairment, relatives, friendships and self-reported depression supplemented in CFAS II. No exercise only measured in those not chair or bed bound.

		*CFAS I*						*CFAS II*					
		Dementia (n = 128)			No Dementia (n = 55)			Dementia (n = 137)			No Dementia (n = 54)		
		n	Weighted %	95% CI	n	Weighted %	95% CI	n	Weighted %	95% CI	n	Weighted %	95% CI
Female		96	77.5	69.1–84.2	38	72.5	52.2–86.4	103	80.1	72.4–86.0	34	66.4	52.5–78.0
Age	≤69	8	5.3	2.6–10.6	1	1.0	0.1–7.3	6	6.2	2.4–15.3	5	7.6	3.1–17.6
	70–74	7	4.5	2.1–9.5	10	10.3	5.0–20.1	3	1.7	0.5–5.5	4	5.8	2.1–15.1
	75–79	10	7.8	4.1–14.4	6	9.2	3.6–21.6	19	12.7	7.8–19.8	7	11.8	5.5–23.5
	80–84	37	27.1	19.9–35.8	16	33.4	17.9–53.5	29	17.7	12.1–25.2	7	11.9	5.6–23.5
	85–89	38	32.0	23.7–41.6	9	25.0	9.9–50.3	37	23.9	17.3–32.2	19	33.5	21.8–47.5
	≥90	28	23.3	16.2–32.2	13	21.1	11.2–36.3	43	37.8	28.9–47.6	12	29.5	17.6–45.0
Married		*9 (56)*	*16*.*6*	8.3–30.3	6	8.9	3.6–20.4	30	21.7	15.0–30.4	4	6.4	2.3–16.5
Years in education	≤9	85	74.2	63.0–82.9	38	65.0	44.8–81.0	60	58.7	48.5–68.2	28	58.0	43.1–71.6
	10–11	15	16.2	9.5–26.3	9	17.4	8.3–32.9	35	27.2	19.4–36.8	10	19.8	10.5–34.2
	≥12	7	9.6	4.3–20.0	7	17.6	6.0–42.0	15	14.1	8.3–22.8	11	22.2	12.3–36.8
See relatives at least weekly		*30 (48)*	*60*.*8*	44.1–75.3	29	57.4	36.8–75.7	77	72.0	61.6–80.5	34	75.0	61.0–85.2
Reported Friendships		*36 (52)*	*62*.*2*	45.7–76.2	38	80.4	65.8–89.8	65	62.6	52.9–71.5	44	81.8	67.7–90.6
Functional impairment	Mild/Moderate	0	0	-	3	13.9	3.4–42.5	*0*	*0*	-	3	7.3	2.2–21.4
	Severe	112	96.6	92.0–98.6	45	82.7	57.3–94.5	*63*	*98*.*6*	90.4–99.8	37	80.3	66.0–89.5
	Overall	112	96.6	92.0–98.6	48	96.6	89.1–99.0	*63 (64)*	*98*.*6*	90.4–99.8	40	87.6	75.4–94.2
Chair/bedbound		*11 (48)*	*22*.*9*	12.4–38.6	13	16.8	8.7–29.7	46	38.9	29.7–49.0	10	21.7	11.7–36.7
No Exercise		-	-	-	-	-	-	*17 (25)*	*71*.*7*	*50*.*4–86*.*3*	*22 (36)*	*62*.*8*	*45*.*2–77*.*6*
Number of health conditions	0	28	21.4	14.8–29.9	6	7.4	2.9–17.3	9	6.8	3.4–13.2	2	3.0	0.7–11.8
	1	23	16.7	11.1–24.2	7	9.7	3.9–22.1	20	15.2	9.4–23.5	4	7.3	2.6–19.0
	2	26	19.1	13.1–27.1	5	18.2	5.1–47.9	28	21.9	14.7–31.4	5	12.1	5.0–26.7
	≥3	51	42.8	33.8–52.4	37	64.8	43.3–81.6	75	56.2	46.4–65.4	43	77.6	63.0–87.6
Self-reported depression		*6 (49)*	*11*.*4*	4.7–24.9	5	5.4	2.0–13.6	*9 (64)*	*11*.*2*	5.4–21.8	8	15.9	7.9–29.5
**Measurements from assessment interview**													
Depression		20	16.3	10.2–24.9	13	18.0	9.4–31.7	13	10.0	5.7–17.0	11	21.5	11.9–35.6
Loneliness		*19 (70)*	*26*.*7*	16.7–39.8	13	36.7	17.8–60.8	*14 (43)*	*30*.*8*	18.3–46.8	19	39.9	26.4–55.1

Italics indicate some non-response (total number of people who responded)

### Assisted living facilities

#### Overall

There was a small decrease in the proportion of the older population living in assisted living facilities between CFAS I (9%) and CFAS II (7%), with a decrease in the proportion of women and an increase in the proportion of those aged ≥80 years (Table 1). There were fewer individuals with lower levels of education and more with higher levels between the two time periods (Table 1). Far more individuals in assisted living facilities were married in CFAS I (27%) compared to CFAS II (16%). Dementia prevalence in assisted living facilities was lower than in long term care in both studies and was relatively stable over time (Table 1, p>0.1). There was a large increase over time in the proportion of individuals reporting three or more health conditions from 52% in CFAS I to 72% in CFAS II. Functional impairment also increased from 48% to 67% between CFAS I and II, and, although the percentage of people who were chair or bed bound remained low, it more than doubled between the two studies (Table 1, p = 0.08). Of those who were not chair or bed bound, 13.4% reported doing no exercise in CFAS II. Self-reported depression and AGECAT diagnosis of depression stayed steady over time (Table 1). Loneliness was consistently less reported than in long term care across both studies but was slightly higher in CFAS II (31%) compared to CFAS I (25%) (p>0.1). Three quarters of assisted living facility residents reported seeing their family at least weekly and more reported having friends (nearly 90%) than in long term care in both studies (both p>0.1).

#### Characteristics by dementia status

Table 3 reports the same characteristics for assisted living facilities by dementia status. In both studies a higher percentage of people with dementia than those without had functional impairment overall (Table 3). Mild to moderate and severe functional impairment increased for those with dementia over time whereas only mild to moderate functional impairment increased in those without dementia (Table 3). Self-reported depression increased between the two studies for people with dementia (4% to 9%) but decreased for people without dementia (18% to 10%). Loneliness increased slightly from CFAS I to CFAS II (Table 1) but was consistently higher in those with dementia and the increase over time mainly came from those with dementia (Table 3). In contrast to this, increases in reported friendships were seen in those with dementia but not in those without (Table 3). Increases in the proportion of individuals with three or more health conditions mainly came from those without dementia which was also consistently higher than those with dementia in both studies (Table 3). AGECAT diagnosed depression was similar in both studies for those with and without dementia (both p>0.1).

**Table 3:** Assisted living facilities demographic percentages in CFAS I and CFAS II split for dementia and weighted for age structure, gender and deprivation in CFAS I and CFAS II and additionally for the two stage design in CFAS I with 95% confidence intervals at baseline. Education and health conditions supplemented by informant interview in CFAS I and CFAS II, functional impairment, relatives, friendships and self-reported depression supplemented in CFAS II. No exercise only measured in those who are not chair or bed bound.

		*CFAS I*						*CFAS II*					
		Dementia (n = 34)			No Dementia (n = 107)			Dementia (n = 35)			No Dementia (n = 442)		
		n	Weighted %	95% CI	n	Weighted %	95% CI	n	Weighted %	95% CI	n	Weighted %	95% CI
Female		28	77.7	57.0–90.2	73	67.7	53.4–79.4	27	78.4	60.2–89.7	272	63.3	58.6–67.7
Age	≤69	3	8.5	2.5–25.1	13	7.7	3.6–15.9	0	0	-	36	7.2	5.2–9.8
	70–74	3	10.0	3.0–28.3	22	23.9	14.4–36.8	3	6.9	2.1–20.7	79	16.0	13.0–19.6
	75–79	3	6.9	2.1–20.7	24	29.5	17.7–44.8	2	3.5	0.8–14.6	87	18.7	15.4–22.6
	80–84	11	31.1	17.1–49.6	24	21.8	12.3–35.5	6	14.4	6.1–30.5	111	25.1	21.2–29.4
	85–89	6	17.8	7.6–36.2	18	13.1	7.1–23.1	15	37.4	21.9–55.9	87	20.6	17.0–24.7
	≥90	8	25.7	12.8–44.8	6	4.1	1.7–9.4	9	37.8	20.2–59.2	42	12.5	9.4–16.5
Married		7	23.0	10.5–43.4	26	26.3	15.8–40.4	6	13.8	5.8–29.5	74	16.0	12.9–19.7
Years in education	≤9	27	84.0	67.2–93.1	89	81.9	69.1–90.1	18	56.3	37.9–73.0	202	47.5	42.8–52.3
	10–11	6	16.0	6.9–32.9	10	12.4	5.7–24.6	12	32.7	18.4–51.1	178	39.3	34.8–44.0
	≥12	0	0	-	6	5.8	2.0–15.3	3	11.1	3.3–31.1	60	13.2	10.3–16.7
See relatives at least weekly		21	83.7	64.2–93.6	79	75.4	62.0–85.2	22	77.2	56.7–89.8	308	75.4	71.0–79.4
Reported Friendships		23	84.2	65.3–93.8	91	87.7	76.4–94.0	31	94.7	83.0–98.5	387	87.8	84.4–90.6
Functional impairment	Mild/Moderate	4	11.2	4.0–27.9	21	13.2	7.8–21.5	6	21.4	9.3–42.0	161	37.8	33.2–42.5
	Severe	21	58.9	40.4–75.2	35	30.1	19.0–44.1	18	66.0	45.6–81.7	111	27.2	23.1–31.8
	Overall	25	70.1	50.6–84.3	56	43.3	30.6–56.9	24	87.4	69.0–95.6	272	65.0	60.3–69.4
Chair/bedbound		2	5.6	1.3–21.8	2	0.9	0.2–3.5	1	3.5	0.4–22.6	12	2.8	1.6–4.9
No Exercise		-	-	-	-	-	-	9	38.4	20.2–60.7	49	11.8	9.0–15.4
Number of health conditions	0	4	10.1	3.5–25.6	5	7.9	2.7–20.7	4	11.0	3.8–27.8	9	2.2	1.1–4.2
	1	9	24.7	12.7–42.5	16	13.3	6.0–26.8	7	17.1	7.8–33.6	43	9.4	7.0–12.5
	2	6	20.5	9.0–40.3	24	24.7	14.7–38.4	7	19.4	8.8–37.6	65	14.7	11.7–18.4
	≥3	15	44.8	28.0–62.8	62	54.2	40.4–67.3	17	52.5	33.9–70.4	318	73.7	69.3–77.7
Self-reported depression		1	3.9	0.5–25.5	21	18.3	10.4–30.3	4	8.9	3.0–23.7	46	10.4	7.9–13.7
**Measurements from assessment interview**													
Depression		3	9.1	2.5–28.1	11	9.5	4.2–20.1	4	9.7	3.4–24.6	52	11.6	8.9–15.0
Loneliness		10	32.1	17.2–51.9	28	24.1	14.5–37.3	11	44.3	25.2–65.4	130	30.1	25.9–34.6

### Community Living

#### Overall

Most people were living in the community in both studies (CFAS I 86%, CFAS II 90%). There was consistently a lower proportion of women in the community compared to both other places of residence (Table 1). The age distribution in the community was consistent between CFAS I and II (Table 1). In CFAS I the majority of those living in the community had ≤9 years of education (74%) but in CFAS II the majority had 10–11 years of education (52%). The proportion of married individuals increased slightly over time (Table 1). Dementia prevalence was similar in both studies (Table 1, p>0.1). 44% of individuals in the community had three or more health conditions in CFAS I increasing to 56% in CFAS II. Functional impairment levels increased slightly between both studies (Table 1). The prevalence of being chair or bed bound in the community was low in both studies and only 5% reported doing no exercise if they were not chair or bed bound (Table 1). There was a slight decrease over time in self-reported depression (p<0.01) and in loneliness, though a slight increase in reported friendships and similar frequency of seeing relatives (Table 1).

#### Characteristics by dementia status

Table 4 gives results for the same characteristics split by dementia status in the community. The proportion with functional impairment was similar in both studies (p>0.1) but was consistently higher and more severe in people with dementia (Table 4). AGECAT diagnosed depression was higher in those with dementia in CFAS I compared to those without dementia but this decreased to the same level in CFAS II (Table 4). The proportion of individuals with dementia and three or more health conditions increased between CFAS I (49%) and CFAS II (62%), this also happened in individuals without dementia but to a lesser extent. More people without dementia reported friendships than people with dementia in both studies (Table 4).

**Table 4:** Community living demographic percentages in CFAS I and CFAS II split for dementia and weighted for age structure, gender and deprivation in CFAS I and CFAS II and additionally for the two stage design in CFAS I with 95% confidence intervals at baseline. Education and health conditions supplemented by informant interview in CFAS I and CFAS II, functional impairment, relatives, friendships and self-reported depression supplemented in CFAS II. No exercise measured only in those who are not chair or bed bound.

		*CFAS I*						*CFAS II*					
		Dementia (n = 164)			No Dementia (n = 964)			Dementia (n = 302)			No Dementia (n = 6750)		
		n	Weighted %	95% CI	n	Weighted %	95% CI	n	Weighted %	95% CI	n	Weighted %	95% CI
Female		102	71.3	61.8–79.2	586	60.4	56.1–64.6	163	58.3	52.2–64.2	3596	54.4	53.2–55.6
Age	≤69	9	3.9	1.9–7.7	276	27.0	23.5–30.8	16	4.9	2.9–8.2	1876	26.2	25.2–27.3
	70–74	18	7.0	4.1–11.7	259	23.2	20.0–26.8	41	11.6	8.4–15.6	1743	24.8	23.8–25.8
	75–79	33	26.0	15.2–40.7	186	23.9	20.1–28.3	67	20.2	15.8–25.5	1441	21.3	20.4–22.3
	80–84	57	34.5	24.2–46.5	146	17.7	14.2–21.7	82	27.3	22.1–33.2	1042	16.4	15.5–17.3
	85–89	39	25.1	15.8–37.5	75	6.8	4.9–9.4	57	19.5	15.0–24.9	490	7.9	7.2–8.6
	≥90	8	3.6	1.7–7.3	22	1.4	0.7–2.6	39	16.7	12.0–22.7	158	3.4	2.9–4.0
Married		64	35.1	25.2–46.5	487	51.9	47.4–56.3	155	52.4	46.2–58.6	4116	59.6	58.4–60.8
Years in education	≤9	138	80.6	63.6–90.8	750	68.7	64.2–73.0	134	46.9	40.7–53.3	1600	25.2	24.2–26.3
	10–11	14	17.4	7.6–35.2	143	18.3	15.0–22.2	120	40.5	34.6–46.8	3569	52.2	51.0–53.4
	≥12	3	2.0	0.6–6.3	69	13.0	9.8–16.9	34	12.6	8.8–17.6	1544	22.6	21.6–23.6
See relatives at least weekly		106	84.7	76.8–90.3	715	75.1	70.7–79.0	214	81.8	76.3–86.3	4927	77.3	76.3–78.4
Reported Friendships		89	72.5	62.0–81.0	762	81.9	78.4–85.0	203	69.7	63.5–75.3	5809	86.1	85.2–86.9
Functional impairment	Mild/Moderate	20	23.1	12.0–39.9	162	16.2	13.2–19.8	45	17.9	13.5–23.3	1283	20.0	19.1–21.0
	Severe	86	47.2	34.9–60.0	160	11.6	9.3–14.5	130	56.6	50.1–62.9	642	10.4	9.6–11.2
	Overall	106	70.4	57.7–80.5	322	27.9	24.2–31.9	175	74.5	68.6–79.6	1925	30.4	29.3–31.5
Chair/bedbound		11	5.7	3.0–10.7	10	0.8	0.3–1.9	23	9.5	6.0–14.8	40	0.6	0.5–0.8
No Exercise		-	-	-	-	-	-	44	24.2	18.5–31.1	257	4.2	3.7–4.7
Number of health conditions	0	16	6.4	3.7–10.8	81	8.2	6.2–10.9	24	7.4	4.9–11.1	468	6.8	6.2–7.4
	1	30	19.6	11.1–32.4	201	22.4	18.9–26.3	39	13.4	9.6–18.4	1068	15.6	14.7–16.5
	2	31	25.4	14.6–40.5	197	21.0	17.6–24.9	54	17.5	13.2–22.7	1458	21.5	20.6–22.5
	≥3	87	48.6	36.5–60.7	485	48.4	44.0–52.8	179	61.8	55.5–67.7	3724	56.1	54.9–57.3
Self-reported depression		14	8.9	4.8–15.9	122	11.3	8.9–14.2	29	12.4	8.6–17.8	483	7.2	6.6–7.9
**Measurements from assessment interview**													
Depression		34	23.5	13.2–38.5	106	7.6	5.8–9.9	30	8.5	6.0–12.0	423	6.3	5.7–6.9
Loneliness		33	29.5	17.4–45.3	200	19.9	16.5–23.7	50	23.8	18.4–30.2	1024	15.8	14.9–16.7

### Sensitivity Analyses

Sensitivity analyses showed that the inverse probability weighted prevalence estimates did not change when either removing long term care from the weights in CFAS II or with the change in classification of “granny flats” from community living in CFAS I to being assisted living facilities in CFAS II.

## Discussion

Changes to policy take time to be implemented and the two decade gap between CFAS I and CFAS II provided sufficient time to see substantial changes. As reported previously, there was an increase in prevalence of dementia within long term care [10]. There has also been an increase in the proportion of individuals reporting three or more health conditions across all places of residence, but evidence from both studies suggested that individuals with dementia in long term care had fewer health conditions than those without dementia. The level of functional impairment remained the same in long term care between studies, being consistently high, whilst in assisted living facilities functional impairment increased considerably.

Both CFAS I and II provide an accurate representation of the older population, their characteristics, and their living situation as they were large, randomly sampled, population based studies from several centres and collecting place of residence was part of the study design. The methods and analyses were identical, including the study diagnosis of dementia by the AGECAT algorithm. However, there were some limitations. CFAS II was conducted in three centres which restricted the CFAS I analysis to these centres too. Some may argue that these areas might not be representative of the whole UK, particularly in terms of service provision which are affected by policies of different local authorities. They do however provide more evidence than exists to date across deprived and affluent settings. Response rates fell between the two studies, in keeping with other population based studies [14, 15]. Weighting was introduced to account for the variability in non-response and as reported before, sensitivity analysis showed findings were robust to this non-response [10]. There was more geographic mobility when entering long term care with 23% of long term care residents having lived in the area for less than 4 years compared to 4% of those in the community (results not shown in tables); long term care was therefore included in the weights in CFAS II. Changes over time meant “granny flats” were included as different places of residence in CFAS II but sensitivity analysis confirmed this did not drive the findings. Informant data were used if data from participant interviews were missing. Although informants will not always report the same information as the respondent [16, 17], the data are a valuable source of information and more useful than larger volumes of missing data. Informants cannot be used to report on subjective outcomes, although informants were used for self-reported depression as this referred to the diagnosis of depression and not feelings of low mood. Reported friendships and seeing relatives also was covered by informant report in CFAS II. Although informants could potentially respond differently so as not to seem inattentive, there were no differences in results when the informant interview was not used to supplement these questions. Unfortunately, even with the use of the informant interview it was not possible to do a direct comparison between the studies for some of the factors due to item non-response.

The reduction of people living in long term care followed the pattern found in the 2011 census [2]. The age and gender distributions found in other studies are similar to the results here [8, 9, 18]. Normally people move to long term care when their community support breaks down due to increasing dependency or loss of support structure. These results are consistent with changes in local authority policies that resulted in a higher level of dependency needing to be reached before public funding for residential care is offered.

Recent studies have reported a lower prevalence of dementia within long term care in the UK in comparison to CFAS II [9, 19, 20], however this could be because they only included individuals with a formal diagnosis of dementia [19, 20]. Others suggest estimates closer to CFAS II although a direct comparison cannot be made as the study diagnosis of dementia was different for each [18, 21].

Reported estimates of functional impairment for people in long term care have varied. This could be due to the binary definition of functional impairment compared to the three point scale used here [22] and also differs between high income countries [22, 23] although this is only reported at one time point rather than looking at a comparison of change over time. Another English study reported similar estimates to CFAS but looked at those aged 85 and above [24]. An increase in functional impairment was reported in long term care before CFAS I but the recent reduction in people moving to long term care may explain the stability observed as levels may have instead increased in assisted living facilities [3]. Another study predicts an increase in the number of those with critical need dependency although this is not in assisted living facilities specifically [24]. If more of those in assisted living facilities become dependent then increased support needs to be made available with implications on policies and services trying to plan their care.

Other studies report on average more than three health conditions in long term care but the percentage estimates from CFAS suggest that these averages could be skewed [20, 25]. In long term care, more people without dementia reported three or more health conditions compared to people with dementia in both CFAS I and II. It seems that individuals without dementia move into long term care because an accumulation of long term conditions brings about irreversible functional impairment. This was supported by the fact that the number of health conditions was higher in people without dementia in assisted living facilities but not in the community. One study from the US reported similar levels of health conditions when comparing the mean in those with and without dementia but as stated above, these estimates could be skewed [26]. Other reports on multiple health conditions that separated for those with and without dementia in long term care from either within the UK or other high income countries do not compare the overall number of conditions in those with and without dementia [8, 9, 25].

The prevalence of depression in long term care increased over the last few decades but the estimates from CFAS were still lower than estimates from other studies which could reflect different methods of ascertainment [18, 27, 28]. These estimates suggest that depression represents a potentially improvable state affecting quality of life.

Few studies have reported on the prevalence of loneliness specifically in long term care. The estimate from CFAS II of reported loneliness was at the higher end of a range given by Oxfordshire Age UK [29], although others have reported higher figures [30]. These findings suggest that for those who are able, loneliness is a major factor even with the presence of other people. Strategies tackling this directly need to be explored to see what works for individuals.

Other measurements previously linked with quality of life have shown here to have positively changed over time. In long term care there was an increase in reports of seeing relatives at least weekly so that in CFAS II it was equal across all places of residence. It was also encouraging to see that, although lower for those with dementia in long term care, reported friendships were high across all places of residence in both studies.

Alongside the decrease in the percentage of older people living in long term care over the past two decades, those that do so are more likely to have dementia, be highly dependent and have a higher number of health conditions than previously whilst those without dementia are physically frail and have multiple health conditions. Over this time the percentage of people in long term care with functional impairment has been steady and close to 100%. The fact that levels of functional impairment have also remained steady in the community but have increased in assisted living facilities could indicate a point when it becomes too difficult or too costly for an individual to live independently. The rising number of older people with high levels of need living in the community could mean that the decline in moving to long term care may have already reached, or be close to, the point of reversal. This is especially important as findings from CFAS I and II predate changes to current reductions in funding settlements and evidence suggests there will be increasing demand for long term care places [24]. The NHS Five Year Forward View focused on the need for integrated care between community, GP, and hospital services [31]. This could help meet the objectives set out by The National Dementia Strategy to improve quality of care for people with dementia in long term care through the development of inspections and explicit leadership for dementia care, possibly commissioning specialist services from community mental health teams [21]. However, receiving care in the community can put pressure on unpaid carers [32–34] and, in addition, there needs to be support in place for those caring for people with dementia. These results have implications for policies on long term care as well as home care including quality of care, health management, cost, and the development of a skilled, caring, and well informed workforce.
