# Correction: Who Lives Where and Does It Matter? Changes in the Health Profiles of Older People Living in Long Term Care and the Community over Two Decades in a High Income Country

**DOI:** 10.1371/journal.pone.0164935

**Published:** 2016-10-12

**Authors:** The PLOS ONE staff

There is an error in the byline for the 7^th^ author. The correct author name should read: on behalf of the Cognitive Function Ageing Studies (CFAS) collaboration. The publisher apologizes for the error.
