# Correction: Correction: Who Lives Where and Does It Matter? Changes in the Health Profiles of Older People Living in Long Term Care and the Community over Two Decades in a High Income Country

**DOI:** 10.1371/journal.pone.0174193

**Published:** 2017-03-14

**Authors:** The PLOS ONE Staff

The name of the author group is incorrect. The correct author group is Cognitive Function and Ageing Studies (CFAS) collaboration. The publisher apologizes for the error.
